# Structure and Interdigitation of Chain-Asymmetric Phosphatidylcholines and Milk Sphingomyelin in the Fluid Phase

**DOI:** 10.3390/sym13081441

**Published:** 2021-08-05

**Authors:** Moritz P. K. Frewein, Milka Doktorova, Frederick A. Heberle, Haden L. Scott, Enrico F. Semeraro, Lionel Porcar, Georg Pabst

**Affiliations:** 1 Institute of Molecular Biosciences, University of Graz, NAWI Graz, 8010 Graz, Austria; 2 Institut Laue-Langevin, 38043 Grenoble, France; 3 BioTechMed Graz, 8010 Graz, Austria; 4 Field of Excellence BioHealth, University of Graz, 8010 Graz, Austria; 5 Department of Molecular Physiology and Biological Physics, University of Virginia School of Medicine, Charlottesville, VA 22903, USA; 6 Department of Chemistry, University of Tennessee Knoxville, Knoxville, TN 37996, USA; 7 Center for Environmental Biotechnology, University of Tennessee, Knoxville, TN 37996, USA; 8 Shull Wollan Center, Oak Ridge National Laboratory, Oak Ridge, TN 37831, USA

**Keywords:** mixed-chain lipids, neutron scattering, X-ray scattering, MD simulations

## Abstract

We addressed the frequent occurrence of mixed-chain lipids in biological membranes and their impact on membrane structure by studying several chain-asymmetric phosphatidylcholines and the highly asymmetric milk sphingomyelin. Specifically, we report trans-membrane structures of the corresponding fluid lamellar phases using small-angle X-ray and neutron scattering, which were jointly analyzed in terms of a membrane composition-specific model, including a headgroup hydration shell. Focusing on terminal methyl groups at the bilayer center, we found a linear relation between hydrocarbon chain length mismatch and the methyl-overlap for phosphatidylcholines, and a non-negligible impact of the glycerol backbone-tilting, letting the *sn1*-chain penetrate deeper into the opposing leaflet by half a CH_2_ group. That is, penetration-depth differences due to the ester-linked hydrocarbons at the glycerol backbone, previously reported for gel phase structures, also extend to the more relevant physiological fluid phase, but are significantly reduced. Moreover, milk sphingomyelin was found to follow the same linear relationship suggesting a similar tilt of the sphingosine backbone. Complementarily performed molecular dynamics simulations revealed that there is always a part of the lipid tails bending back, even if there is a high interdigitation with the opposing chains. The extent of this back-bending was similar to that in chain symmetric bilayers. For both cases of adaptation to chain length mismatch, chain-asymmetry has a large impact on hydrocarbon chain ordering, inducing disorder in the longer of the two hydrocarbons.

## 1. Introduction

As the main structural constituents of biological membranes, glycerophospholipids and sphingolipids occur in a large variety of species, differing in their hydrophilic heads, hydrophobic tails and backbone structure. A considerable fraction of the most abundant double-chained membrane lipids exhibit distinct compositional differences of their hydrocarbons [1,2]. Particularly, combinations of a saturated and an unsaturated chain are very common for glycerophospholipids and are therefore widely used in membrane mimics. Some of these, and in particular monounsaturated phosphatidylcholines (PCs) such as palmitoyl oleoyl PC (POPC) or stearoyl oleoyl PC (SOPC) are therefore, well characterized in their fluid phase structures [3]. In contrast, saturated phospholipids with mixed chain lengths are much less abundant and hence less frequently studied. Large chain length asymmetries including long, saturated chains are, however, frequent in sphingolipids, such as, e.g., sphingomyelin. Sphingomyelin contains a sphingosine backbone of 18 carbons and an acyl chain, which can largely vary in length. Its chain asymmetry and heterogeneity have been shown to impede the formation of liquid-ordered domains in mixtures with cholesterol [4], which might be due to hydrocarbon packing stresses caused either by a penetration of the longer hydrocarbon chain into the opposing leaflet (interdigitation) or by bending the chain back into its host leaflet. Further hydrocarbon chain interdigitation has been also implied in the transleaflet coupling of asymmetric lipid bilayers [5-7].

In order to explore the effects of a hydrocarbon chain interdigitation versus chain backward bending, we focused on the chemically well-defined stearoyl myristoyl PC (SMPC), myristoyl stearoyl PC (MSPC) and palmitoyl myristol PC (PMPC). These lipids melt close to physiological temperatures, but their melting temperature (*T*_*m*_) strongly depends on the degree of chain length asymmetry [8]. Interestingly, thermotropic data for SMPC, MSPC and dipalmitoyl PC (DPPC) suggest that the *T_m_* is highest for equal chain lengths, which occurs however not for DPPC, but for a hypothetical lipid with an *sn2*-chain that is about 1.5 carbon units longer than the *sn1* chain. This is usually attributed to the ester bonds that link the acyl chains to the glycerol backbone, which causes an effective tilting of the glycerol backbone with respect to the bilayer central plane [9,10]; see supplementary Figure A1 for lipid structure. On the other hand, this suggests that the hydrocarbons of the DPPC in the lamellar gel phase are slightly interdigitated. This has indeed recently been confirmed by experiments [11]. In addition to indications of the non-equal location of terminal methyl groups in liquid-ordered domains from NMR experiments [12], studies of such effects in the physiologically more relevant lamellar fluid phase are currently missing, but needed to address the aforementioned issues of hydrocarbon-mediated transleaflet coupling.

We therefore studied the fluid lamellar phases of SMPC, MSPC and PMPC using small-angle X-ray and neutron scattering (SAXS/SANS) experiments, exploiting their different contrasts to enhance structural fidelity [13]. In particular, we jointly analyzed scattering data in terms of compositional modeling, applying a slightly modified version of the well-known scattering density profile (SDP) model [14]. The advanced SDP model in combination with the separated form factor technique [15] allowed us to also include scattering intensities at very low scattering vectors and led us to introduce a hydration layer in the lipid’s headgroup region. The new model was validated against DPPC and confirmed previously reported structural parameters. We consecutively focused on the fluid structures of SMPC, MSPC and PMPC and also included monounsaturated POPC, SOPC and milk sphingomyelin (MSM), which is a natural lipid extract with high chain length asymmetry.

For the fluid phase lipids, we found a large decrease in the lipids’ backbone tilt compared to the gel phase, corresponding to a length difference of about 0.5 carbon units between *sn-2* and *sn-1* chains. Moreover, hydrocarbon chain overlap linearly depends on the chain length mismatch for all studied lipids. All-atom molecular dynamics (MD) simulations further helped to disentangle interdigitated from backward-bending hydrocarbons. Interestingly, we found that close to the lipids’ backbone, the bending back of hydrocarbons into their host leaflet occurs more frequently than interdigitation from the opposing leaflet. This suggests that the effects of backward-bent hydrocarbons on lateral pressures dominate those of interdigitated hydrocarbons.

## 2. Results and Discussion

### 2.1. Introducing a Headgroup Hydration Shell in the Scattering Model for Lipid Bilayers

The SDP model simultaneously accounts for small-angle neutron and the X-ray data (SANS/SAXS) of lipid bilayers thus enabling a unique combination of the different contrasts offered by the two techniques (see, e.g., [13]). The very backbone of the SDP model is a parsing of the trans-bilayer structure into quasimolecular fragments, based on geometrical considerations [16] and MD simulations [14]. This leads to a representation of the membrane structure in terms of Gaussian-type volume probability distributions (Figure A1). The SDP technique has been highly successful in reporting the high-resolution membrane structures of numerous glycero- and sphingolipids [3,17-21], including also polyunsaturated phosphatidylcholines [22].

**Figure 1.**
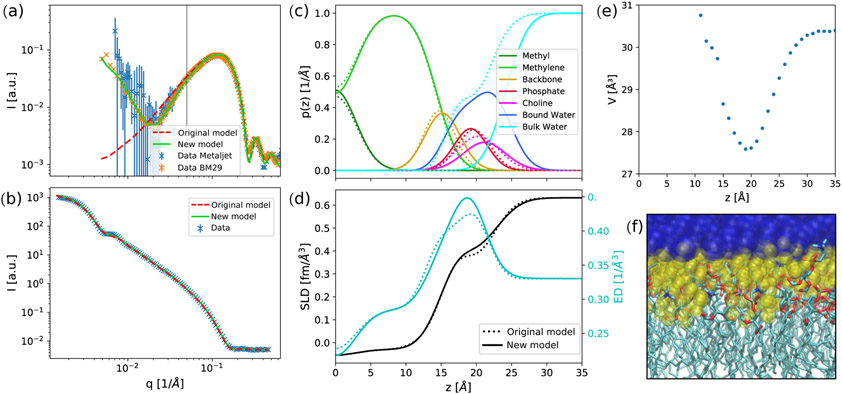
Comparison of the present and original SDP-models [3] for DPPC at 50 °C. The two models mainly show differences in the low-*q* region of SAXS (**a**), whereas they overlap in the case of SANS in 100% D_2_O (**b**). The vertical black line in (**a**) marks the lower limit of the accessible range in the original study. (**c**) shows volume probability distributions *p*(*z*) of the lipid moieties through the bilayer profile. The resulting neutron SLD (black) and electron density profiles (cyan) are drawn in (**d**). Dashed lines correspond to the original, solid lines to the new model. MD-simulations confirm the presence of higher-density water around the headgroup region, where the volume-per-water molecule is decreased by up to 10% (**e**). This effect is schematically illustrated in a simulation snapshot of a DPPC bilayer (**f**) where bulk water is shown in blue (with *z* > 25 Å) and hydration water in yellow (with *z* < 25 Å). Lipids are drawn in a licorice representation with carbons in cyan, nitrogen in blue, phosphate in tan and oxygen in red.

We first implemented the SDP model for a spherical-shell bilayer (i.e., a vesicle) using the separated form factor method [15], which extended the analysis to previously unconsidered low scattering vectors *q* (see Section 4.3 and Appendix A) and performed a test on the benchmark-lipid DPPC. Using published parameters [3], the model fits very well to the SANS data herein, but not to the low-*q* region in SAXS (Figure 1a,b). In particular, the SAXS intensity minimum at *q* ~ 0.02 Å^−1^ is completely missed by the fit, while a good agreement is obtained for *q* > 0.1 Å^−1^, i.e., the *q*-range reported previously [3]. We also measured an independently prepared sample of DPPC using a SAXSpoint laboratory camera. Although these data are intrinsically more noisy, particularly at a low *q*, they clearly agree with synchrotron data and demonstrate that the mismatch of the previous data modeling is a salient feature. Fits to this region have, however, been attained by other models, which unlike SDP, do not depend on the specific composition of the lipid bilayer [23,24]. This indicates that the solution might be an additional degree of freedom in the scattering length density (SLD) profile. Indeed, we found that increasing the contrast in the headgroup region, e.g., by decreasing the headgroup volume, drastically improves the agreement to low-*q* SAXS-data, while having no significant impact on the neutron form factor (data not shown). Note that a similar approach was reported in [25]. An alternative and physically realistic way to do this is to account for the layer of bound water molecules (Figure 1c,d). In this model, we assumed that the water molecules surrounding the polar headgroup take up a more ordered structure than in the bulk, leading to a higher density in this region. Hydration shells of this kind are extensively used for SAXS data analysis of protein solutions [26,27] and have also been predicted for lipid membranes [16]. We implemented hydration water using an error function that adds one layer of more dense water to the water accessible groups of the lipid bilayer as detailed in Section 4.3 and Appendix A. Our fit estimates the water density in this shell to be 3% higher than in the bulk, which agrees with previous reports on hydration shells for proteins or nucleic acids [26,27]. This increased water density between the headgroups can also be found in all-atom MD simulations (Figures 1e,f and A5), where the volume of water molecules near the lipid headgroups decreased by up to 10% compared to the bulk value.

In achieving the fits shown in Figure 1a,b, we also tested for overfitting or parameter correlations. The SDP model relies on a rather high number of adjustable parameters (i.e., 12 to describe the membrane structure) compared to simpler models using slabs [28] or Gaussian distributions [29]. The high number of adjustable parameters is mostly due to the limited available information about the volumes and structures of the individual moieties in the lipid, which are hardly experimentally accessible and can only be estimated from scattering studies and simulations [30]. Previous studies applying the SDP model led to no obvious temperature or composition-dependent trends for several parameters, especially for those describing the headgroup (*σ_CG_, σ_PCN_, D*_*H*1_) and the volume fractions (*R_CG_* = *V_CG_/V_H_, R_PCN_* = *V_PCN_/V_H_, r* = *V*_*CH*3_/*V*_*CH*2_, *r*_12_ = *V_CH_/V*_*CH*2_) [3,20]; see Tables A2 and A3 for a list of all SDP parameters.

**Figure 2.**
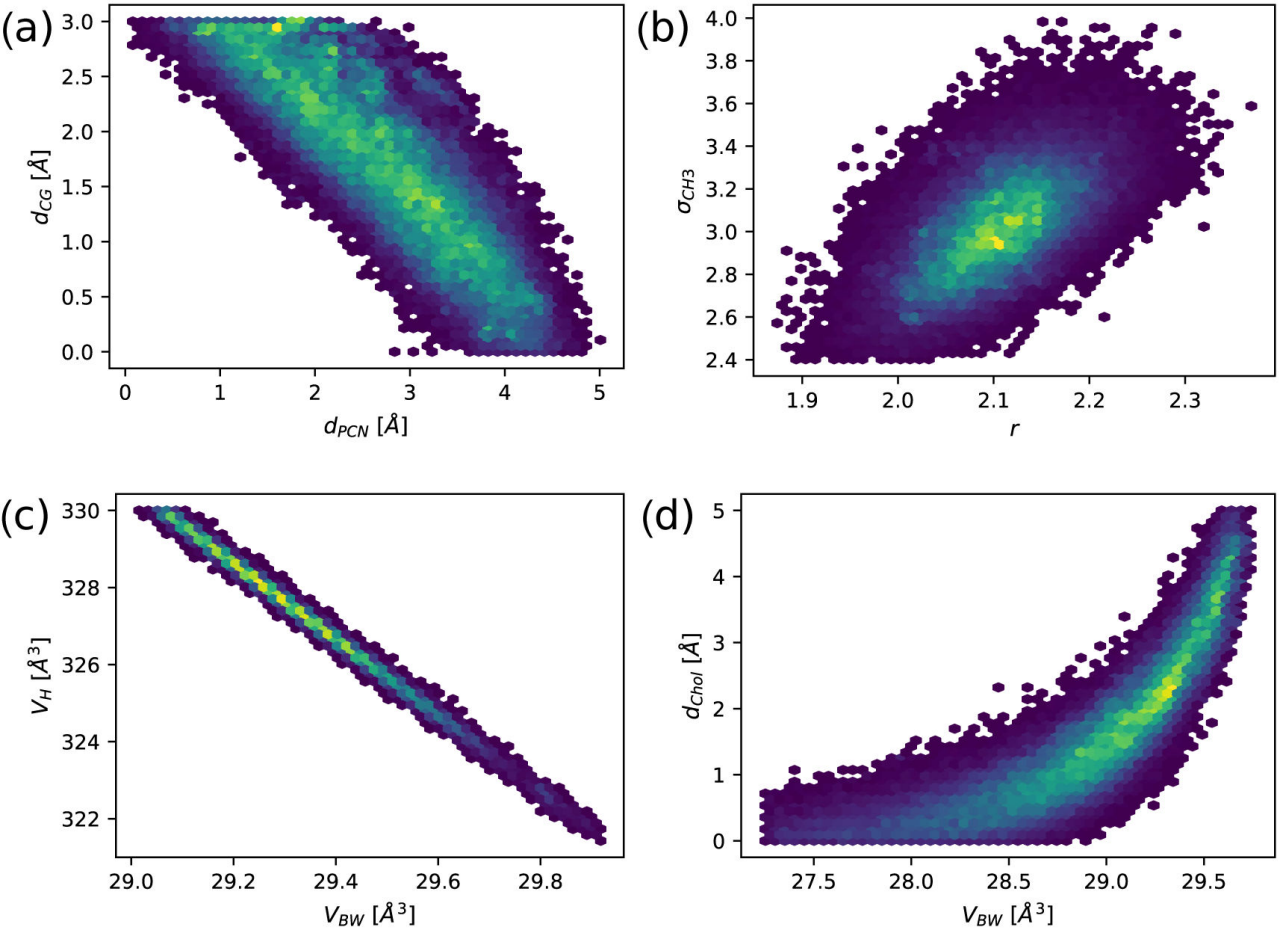
Exemplary parameter correlations in the joint SAXS/SANS-analysis of DPPC vesicles, visualized by MCMC sample histograms. Colored spots correspond to Monte Carlo samples: the brighter the color, the more the samples are contained in the point, thus corresponding to higher probability: (**a**) shows the correlation between the positions of the carbonyl-glycerol and the phosphate group; (**b**) between terminal methyl relative volume *r* and distribution width *σ*_*CH*3_; (**c**) between volume per bound water molecule *V_BW_* and headgroup volume *V_H_* (with constant headgroup structure); and (**d**) between *V_BW_* and the position of the choline-CH_3_ group (with constant *V_H_*).

Parameter correlations were analyzed using a Markov chain Monte Carlo (MCMC) approach as described in Section 4.3 (see also [31]). MCMC provides the probability density profiles of the used model parameters and, if plotted in two dimensions, correlations between them (Figure 2). Plateaus of high probability as seen in, e.g., (Figure 2c), suggest strong correlations, meaning that the quality of the fit will only change minimally if one moves along iso-probability regions. Small differences in the experimental noise can therefore lead to large changes in these parameters, making the estimates of the most likely value (or global minimum) less reliable. In our case, we observed strong correlations between headgroup parameters, such as the positions of carbonyl-glycerol and phosphate groups (Figure 2a). Furthermore, the volume fractions (*R_CG_, R_PCN_, r*) are very flexible parameters insofar that they correlate with the standard deviations of their respective Gaussians (*σ_CG_, σ_PCN_*, *σ*_*CH*3_). Figure 2b shows for example the correlation between *r* and *σ*_*CH*3_. In the following, *σ*_*CH*3_ will be one of our parameters of interest. Therefore, we decided to fix the volume of the CH3 group, along with those of the other moieties to the values recently published in [30] (see Tables A2 and A3), to maximize the comparability between different lipids. This also reduces the number of adjustable parameters for the trans-bilayer structure by three (four in the case of mono-unsaturated lipids) compared to previous studies. We also fixed *σ*_*CholCH*_3__ = 3 Å, as has been done before [3], and *σ*_*CH*2_ = 2.5 Å.

Figure 2c also shows how the introduction of the hydration shell is in fact an alternative to varying the volume of the headgroup *V_H_*. The volume per bound water molecule *V_BW_* is linearly correlated with *V_H_*, if we keep the headgroup structure constant. Varying either of them is thus a valid approach to increase the headgroup SLD. We chose to include the hydration shell in order to conform to published values for the volumes [30]. Additionally, if we keep the headgroup volume constant (*V_H_* = 328 Å^3^), *V_BW_* correlates with the width of the headgroup and thus the number of bound water molecules (shown by the correlation between the distance phosphate to choline *d_Chol_* and *V_BW_* in Figure 2d). The distribution shows the highest probability density between *V_BW_* = 29.0–29.5 Å^3^ for *V_BW_*, which also leads to a physically realistic range of distances *d_Chol_*. We chose *V_BW_* = 29.3 Å^3^, which is at the peak of the distribution.

Despite the improved fit of SAXS data at *q* < 0.1 Å^−1^, we observed only minor changes in membrane structural parameters (Table A2). This can be expected due to the excellent agreement of the previous SDP model for *q* > 0.1 Å^−1^, i.e., for scattering vectors probing distances in the order of the membrane thickness and below. The newly introduced hydration shell gives us an estimate of the number of bound water molecules per lipid. Note that this is not an explicit fitting parameter, but is defined by the integral over the water volume probability density function within the Luzzati thickness, as has been in detail described in [32]. The number of bound water molecules we obtained varied between 9.6 and 12.8 for saturated PCs and MSM, and was about 16 for the more loosely-packed monounsaturated PCs. These numbers agree roughly with previously published values [32,33]. However, there is a wide spread in measured values, mostly due to varying definitions of *n*_*w*_. Furthermore, in our case we attribute a large uncertainty to these values, as it is strongly influenced by the choice of other parameters as discussed above.

### 2.2. Membrane Structure and Interleaflet Hydrocarbon Partitioning

In the next step, we applied our modified SDP anaylsis to various chain-asymmetric PCs as well as the highly asymmetric milk-sphingomyelin extract (average acyl chain length: C22:0). Fits and all parameters are reported in the appendix, in Figures A2 and A3 and Tables A2 and A3. High-resolution structural data for POPC and SOPC were detailed previously [3]. Again, we find no substantial modifications to reported structural details upon the application of our model. To the best of our knowledge, structural details for MSPC, SMPC, PMPC and MSM have not been reported previously, however. Notably, we found that the area per lipid, *A*, of all four lipids is very similar and that *A* of MSPC, SMPC and PMPC agrees within experimental uncertainty with the *A* of DPPC. This demonstrates that chain-asymmetry has no major influence on the general packing of these lipids within the bilayer in the biologically most relevant lamellar fluid phase far above the melting transition. Substituting the *sn2*-hydrocarbon with an oleoyl chain significantly increases *A*, in agreement with [3]. The thickness of the bilayer, *D_B_*, and the thickness of the hydrocarbon chain region, 2*D_C_*, in turn, varies between MSPC, SMPC, PMPC and MSM according to the total number of methylenes. Interestingly, *D_B_* = 40.3 Å for DPPC, MSPC, and SMPC, suggesting that the overall membrane thickness depends for saturated hydrocarbons only on the average number of carbons per chain and is not even influenced by the extreme acyl chain asymmetries of MSPC and SMPC. Note also that the slightly different 2*D_C_* values for these three lipids are equal within experimental resolution.

Several fluid phase structures of sphingomyelins have been recently published [21,34], namely palmitoyl-sphingomyelin (PSM), stearoyl-sphingomyelin (SSM) and egg yolk-sphingomyelin (ESM). In both studies, the structure of PSM was measured at 45 °C; the reported areas per lipid differ, however, possibly due to the different experimental approaches (X-ray surface diffraction on stacks of bilayers vs. SAXS/SANS on vesicles). For ESM, a natural lipid mixture such as MSM, but with PSM as its main constituent and the same structure as for PSM was measured [34], suggesting that hydrocarbon chain heterogeneity does not induce a significant disorder in the chain region. For SSM, however, the reported *A* = 62.5 Å^2^ is considerably higher than the one for PSM [21]. Our result for MSM is again higher (*A* = 64.8 Å^2^), using a similar methodology as reported in [21]. The lateral packing density of sphingomyelin might therefore be directly related to the (average) length of its acyl-chain: PSM/ESM (16:0) < SSM (18:0) < MSM (22:0). Bilayer thickness and terminal methyl overlap are higher for MSM than for the other published lipids, which is expected, again due to its longer acyl chains.

**Figure 3.**
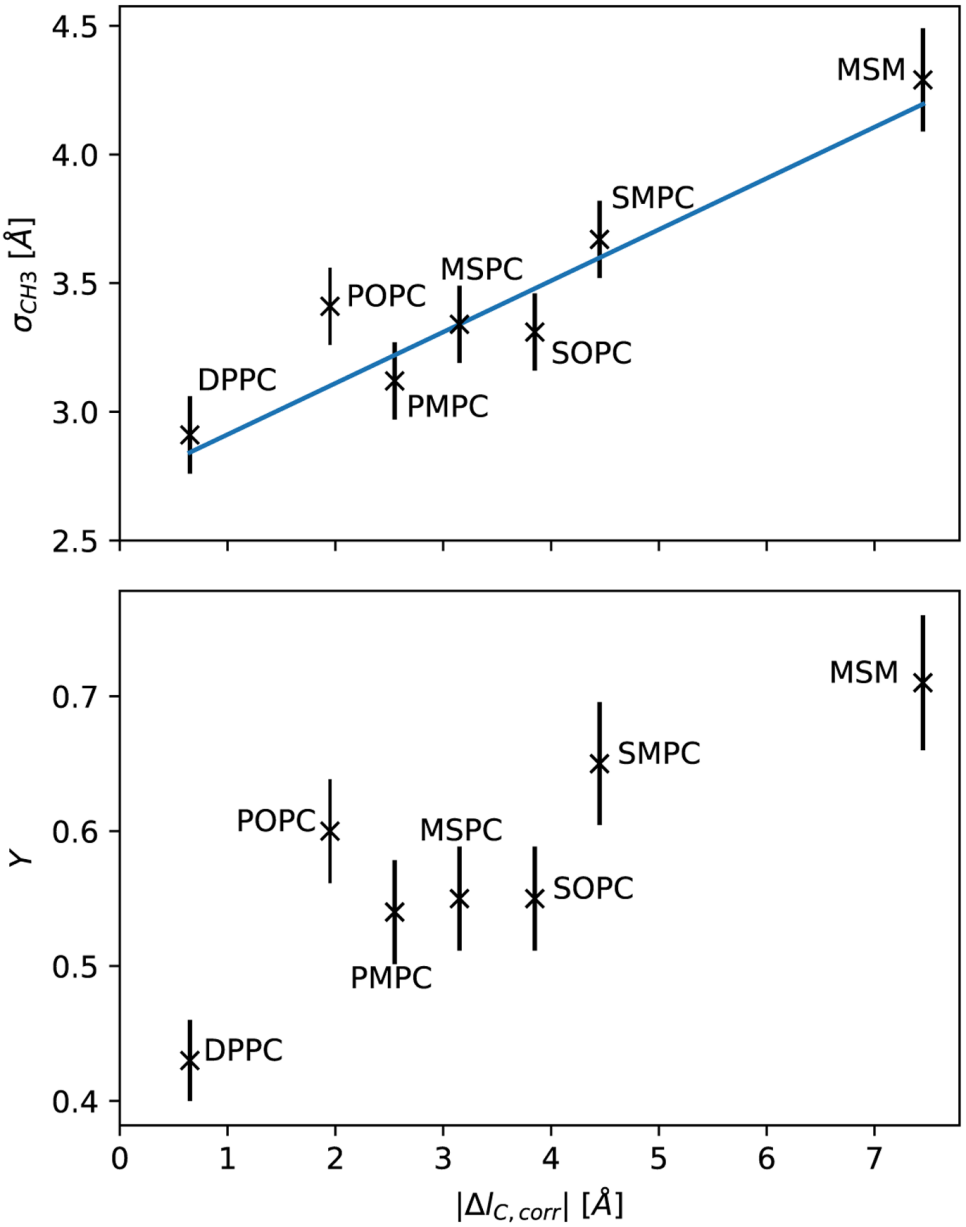
Standard deviations *σ*_*CH*3_ of the Gaussian volume distributions of the terminal methyl groups (**upper** plot) and the relative interdigitation parameters (**lower** plot), plotted over the corrected chain length mismatch ∣Δ*l_C,corr_*∣ of the respective lipids. The upper plot contains a linear regression according to Equation (A1). *σ*_*CH*3_ over uncorrected values ∣Δ*l_C_*∣ are shown in Appendix D Figure A4.

In the following we focus on the hydrocarbon chain interdigitation, which can be expected to be significant given the chain asymmetries of the presently studied lipids. Interleaflet interdigitation may, however, also arise from the specific backbone structure of glycerophospholipids, where the ester bonded hydrocarbon at *sn2* protrudes less into the bilayer core even at nominally equal chain length [10]. Here, we use the width of the terminal methyl group, *σ*_*CH*3_, as a measure for hydrocarbon chain interdigitation. *σ*_*CH*3_ varied significantly for the different lipids studied (Table 1). In order to derive a possible correlation between chain asymmetry and *σ*_*CH*3_, we define the chain length mismatch Δ*l_C_* := *l_C_*(*sn*1) – *l_C_*(*sn*2). Furthermore, we estimated Δ*l_C_* by assuming *l_C_* to be equal to the half-hydrophobic thickness *D_C_* of the corresponding chain-symmetric lipid bilayers (see Table A5). Figure 3 presents the resulting dependence of *σ*_*CH*3_ on Δ*l_C_*. We observed a nearly linear increase in hydrocarbon overlap with increasing chain length mismatch.

SMPC and MSPC possess a priori the same absolute value of chain length mismatch. In this case, it is, however, important to take the well-known tilting of the glycerol backbone [9] into account, which effectively lengthens the *sn1* and shortens the *sn2* chain. We therefore introduce a correction *d_tilt_* on the chain length-mismatch (Equation (1)):

(1) $$\Delta l_{C,corr}≔l_{C}(sn1)-l_{C}(sn2)+d_{tilt}$$

We estimate its value by assuming a linear relation between the corrected, absolute chain length mismatch ∣Δ*l_C,corr_*∣ and *σ*_*CH*3_. In order to evaluate the most likely value for *d*_*tilt*_, we use an iterative approach, alternately optimizing:

(2) $$\sigma_{CH3}=k|\Delta l_{C,corr}|+\sigma_{CH3}^{sym},$$

and Equation (1). Here, *k* is the slope and $\sigma_{CH3}^{sym}$ is the terminal methyl width of a hypothetical lipid of equally long chains; for details, see the pseudocode Algorithm A1.

The result is shown in the upper panel of Figure 3, with the value *d_tilt_* = 0.48 Å. In terms of chain length dependence on the number of carbons (Table A5), this corresponds to about half the length of a CH_2_-segment. The parameters of the linear fit result in *k* = 0.20 and $\sigma_{CH3}^{sym}=2.75\;Å$. The chain overlap thus rises only slowly with the chain length mismatch (20% of its length), which fits into a bilayer picture of fluid hydrocarbon chains, not directly pointing towards the center, but significantly diverted and/or bent. Note that our analysis indicates that even DPPC has some inherent hydrocarbon interdigitation.

### 2.3. Quantifying Hydrocarbon Chain Overlap Relative to the Hydrophobic Thickness

The standard deviation of the Gaussian accounting for the terminal methyl groups *σ*_*CH*3_ gives a measure for hydrocarbon chain interdigitation or, more precisely, the terminal methyl dislocation. However, in some cases, it might be helpful to describe this quantity relative to the thickness of the hydrocarbon layer to estimate its effect on chain disordering. We therefore introduce the dimensionless parameter *ϒ* and connect it to the SDP model, by defining the state *ϒ* = 0 (no chain overlap) when the volume probability density of the CH_3_-groups reaches one at the bilayer center. This is the case for $\sigma_{CH3}^{0}=2V_{CH3}∕(\sqrt{2\pi }A)$. Furthermore, we define the state *ϒ* = 1 by 3*σ*_*CH*3_ = *D*_*C*_, representing a smeared-out state, where the CH_3_ volume is distributed over the whole hydrocarbon region (fully interdigitated). This leads to the definition:

(3) $$\Upsilon ≔\frac{\sigma_{CH3}-\sigma_{CH3}^{0}}{D_{C}∕3-\sigma_{CH3}^{0}}.$$

The extreme states (*ϒ* = 0, 1) are most likely purely theoretical. $\sigma_{CH3}^{0}$ is around 1.4 Å for the studied lipids, while results from Section 2.2 suggest that *σ*_*CH*3_ ≥ 2.75 Å for PC-lipids. Moreover, the *σ*-values of other molecular groups also lie far above this value, suggesting that overall fluctuations of the molecules will not permit localization to such an extent. On the other hand, for *ϒ* approaching 1, the probability distribution of the CH_3_ group might no follow a Gaussian shape. In intermediate cases, as for systems used in this study, *ϒ* could mark a major characteristic of a bilayer. Here, our results suggest that the relative dislocation of the chain termini also monotonously increases with hydrocarbon chain mismatch (Figure 3), and can reach up to ~70% of hydrocarbon chain thickness. POPC, interestingly, does not fit into this picture, having within experimental uncertainty a relative chain overlap similar to that of SOPC or SMPC. This is most likely a signature of the unsaturated hydrocarbon, which increases due to its kink at the *cis* double bond the width of the distribution of the terminal CH_3_.

### 2.4. Chain Interdigitation and Back-Bending in Simulated Systems

**Figure 4.**
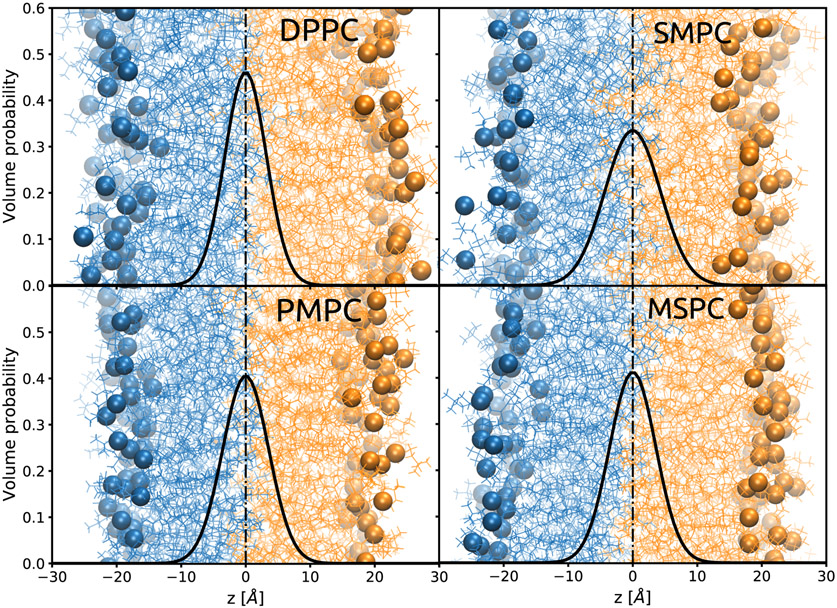
Snapshots of MD simulations for saturated phosphatidylcholines. Spheres mark the positions of phosphorus. The overlaid graphs represent the volume probability distributions of the CH_3_ groups, summed over all lipids in the bilayer.

From our experiments, we were not able to distinguish between lipids in the inner in and outer leaflets. Hence, broadening of the CH_3_-Gaussian could be either caused by interdigitation or by the back-bending of the longer hydrocarbon chain. In order to clarify this issue, we performed MD-simulations on DPPC, MSPC, SMPC, PMPC and dimyristoyl PC (DMPC) to gain access to details in the behavior of the hydrocarbon chains at the bilayer center. Simulation snapshots and the overall volume probability distributions of terminal methyl groups of DPPC, MSPC, SMPC, PMPC are shown in Figure 4. In all cases, the CH_3_ distributions are centered in the middle of the bilayer, although their widths are broader than our experimental values (Table A2). However, the trend over the chain length mismatch agrees with our experimental observation. The snapshots additionally show a significant number of chains penetrating deeply into the opposing leaflet for MSPC, PMPC and SMPC. Overlaid are the volume probability distributions of the terminal methyls, which result from fluctuations of both the individual chains and whole lipid molecules (protrusions).

**Figure 5.**
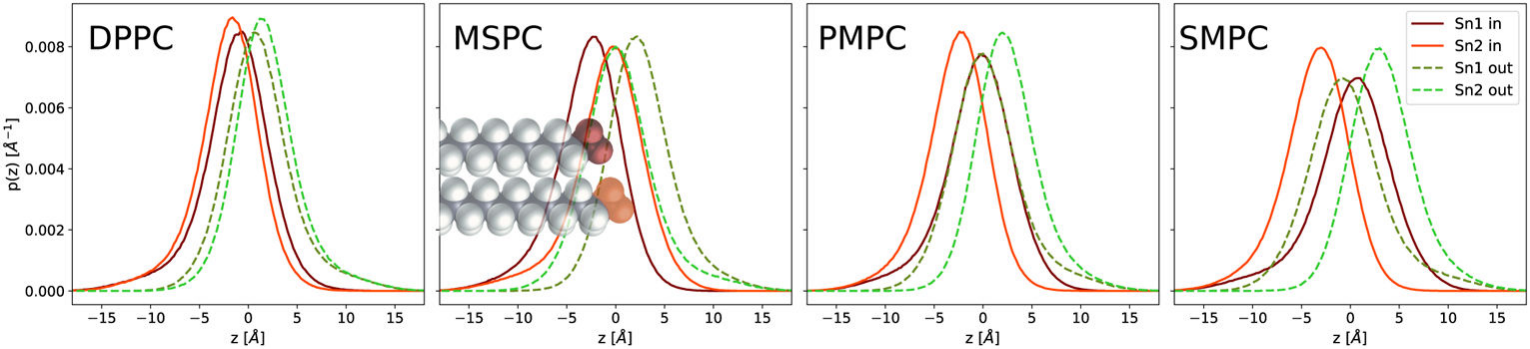
Number probability distributions *p*(*z*) from MD simulations of the terminal methyl groups, separately plotted for lipids from the inner (**left**) and outer (**right**) leaflet, as well as for *sn*1- and *sn*2-chains.

A closer look into the shape of the CH_3_ distribution functions reveals that they actually decay slower than Gaussians (Figure A6). Separating the distribution into contributions from *sn1* and *sn2*-chains, from inner and outer leaflet (Figure 5) leads to further insight. In particular, one can see that the deviation from a bell-shaped function is connected to the shape of the distributions of the individual chains, which are slightly asymmetric with a tailing to the back towards their headgroups. This tailing is equally present for DPPC and thus not a consequence of chain asymmetry. However, while for DPPC all methyl groups clearly have the peaks of their distributions in their own leaflet, the distributions of the shorter chains from inner and outer leaflets are well separated for MSPC, PMPC and SMPC, while the long chains overlap much more. In the case of MSPC and PMPC, the long chain distribution functions from opposing leaflets almost perfectly overlap in the center of the lipid bilayer and only deviate in the tailing toward the headgroup region. This suggests that there is a balance between hydrocarbon interdigitation and back-bending in the center of the membrane, while contributions from backward bent chains dominate over interdigitated hydrocarbons when moving closer to the glycerol backbone. This asymmetric part accounts for 8% of the total area of the distribution (Figure A6). This can be alternatively visualized by plotting the fraction of lipids with their methyl termini located above a certain distance from the center of the bilayer (Figure A7). In the case of SMPC, the long chains penetrate deeper, with the maxima of their distributions in the opposing leaflet.

An interesting consequence of the prevalence of contributions from back-bent hydrocarbons further away from the bilayer center becomes clear considering that packing defects typically have larger effects on the lateral pressure profile, if they occur closer to the glycerol backbone [35]. That is, even if we do find similar lipid areas for DPPC, SMPC, MSPC, and PMPC, their stored elastic energies may differ significantly and will be dominated by the back-bent hydrocarbons, not by the interdigitating ones.

**Figure 6.**
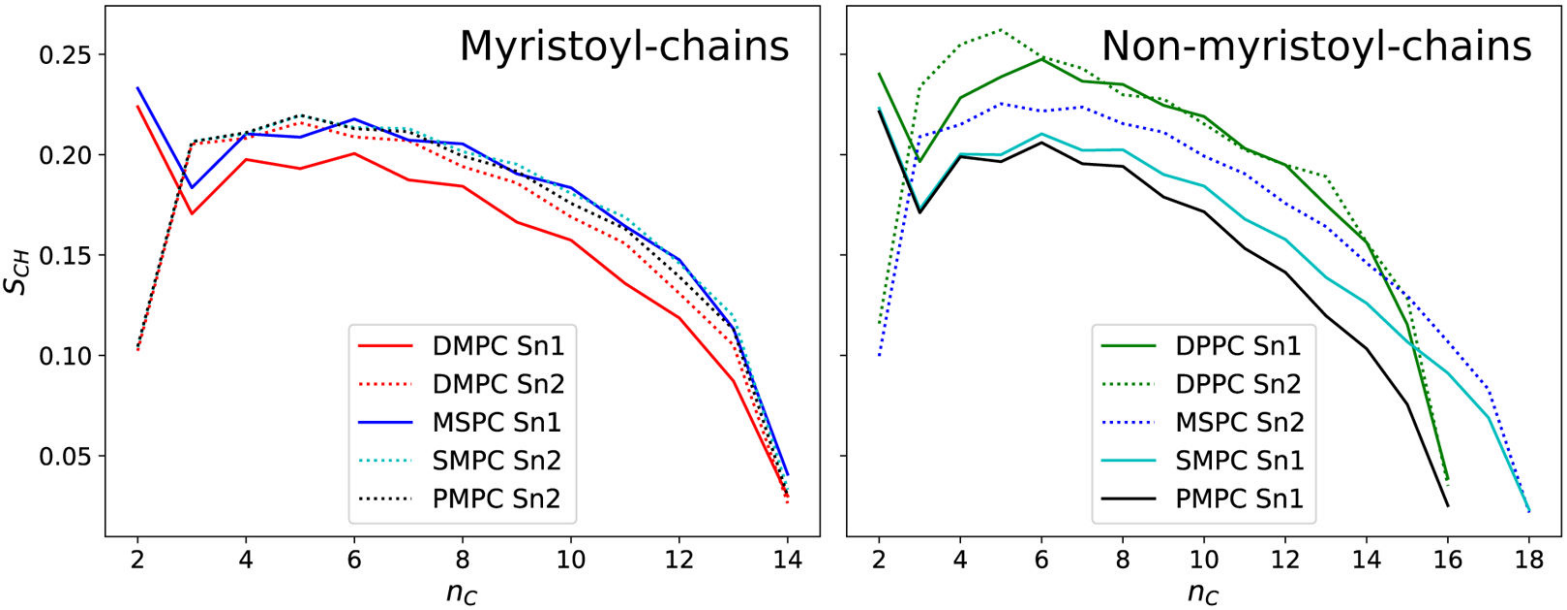
Orientational order parameters *S_CH_* from MD-simulations for individual lipids and chains.

Another effect of the hydrocarbon chain mismatch can be seen in the orientational order parameter *S_CH_* of the hydrocarbons, which was also derived from MD simulations (Figure 6). This dimensionless number represents the average orientation of the respective C─H bonds relative to the bilayer normal [36] and approaches 1 for perfectly ordered chains. Hydrocarbons are labelled by the number *n_C_*, starting with 1 at the ester bond. In the case of chain-symmetric lipids, the strength of the attractive van der Waals interactions between the hydrocarbon chains increases with chain length, leading to a higher ordered state, as can be seen in the example of DMPC (14 carbons/chain) and DPPC (16 carbons/chain). If there is a chain length mismatch, however, the longer chain lacks its direct neighbor at its tip, decreasing its order. In fact, order parameters of the longer chains in MSPC, SMPC and PMPC are close to the ones of DMPC for low *n_C_* and well below those of DPPC. Again, we see a difference between MSPC and SMPC: due to the glycerol-tilt, the 18:0 chain in MSPC has a lower effective length difference to its 14:0 chain and is therefore more ordered than in SMPC. On the other hand, the behavior of the short myristoyl-chain is almost identical for all lipids, as they all have a long neighboring chain to optimize van der Waals’ interactions. Solely the *sn1*-chain in DMPC, again being longer than its *sn2* due to the glycerol-tilt, has slightly lower order parameters.

## 3. Conclusions

We report trans-bilayer structural profiles of free-floating large unilamellar vesicles containing several chain-asymmetric PCs as well as milk sphingomyelin. Additionally, we introduced a shell of hydration water into the well-established SDP model, which allowed us to model low-*q* SAXS-data conserving previously reported lipid headgroup volumes. For fully saturated PCs, we observed no significant effects on the overall bilayer structure resulting from the chain asymmetry, except for the overlap of their terminal methyl groups in the membrane center. This overlap displays a linear dependence on the length difference between both acyl chains, if one considers the tilt of the glycerol backbone. We found that the tilt elongates the *sn1*-chain by 0.48 Å, which is about one third of the value previously reported for gel phases [8]. For PCs with a saturated and an unsaturated chain, we find a poorer agreement with the linear relation between chain length difference and hydrocarbon overlap, which might be a consequence of the kink induced at the double bond. MSM in turn is well described by the model and shows, as expected, the highest hydrocarbon chain overlap of all studied lipids. It has, however, a lower packing density than fully saturated PCs, which agrees with other recent studies, suggesting that long acyl chains lead to a lower packing density in the case of sphingomyelins.

Using MD simulations, we found that every chain, which does not have an equally long or longer direct neighbor, is significantly more disordered—not only at its tip, but over the whole chain length. Moreover, close investigation of the positions of the methyl groups revealed that chains are not symmetrically distributed around a mean position, but have a higher fraction of chains bending back towards their own headgroup. Since membrane elasticity is more affected by packing defects close to the lipids’ backbone, this suggests a dominating role of back-bent over interdigitated hydrocarbons in any membrane-mediated effect related to lateral pressure changes in this region of the bilayer.

## 4. Materials and Methods

### 4.1. Lipids, Chemicals and Sample Preparation

All lipids were purchased in the form of powder from Avanti Polar Lipids (Alabaster, AL, USA) and used without further purification. Chloroform and methanol (pro analysis grade) were obtained from Merck KGaA, Darmstadt, Germany. Lipid films were prepared by dissolving weighted amounts in organic solvent chloroform/methanol (2:1, vol/vol) followed by evaporation under a soft N_2_ stream and overnight storage in a vacuum chamber. The dry films were hydrated with ultrapure H_2_O, D_2_O or a mixture of both, and equilibrated for one hour at 50 °C followed by 5 freeze-and-thaw cycles using liquid N_2_ and intermittent vortex-mixing. Large unilamellar vesicles (LUVs) were obtained by 51 extrusions with a hand held mini extruder (Avanti Polar Lipids, Alabaster, AL, USA) using a 100 nm pore diameter polycarbonate filter. Vesicle size and polydispersity was determined via dynamic light scattering using a Zetasizer NANO ZS90 (Malvern Panalytical, Malvern, UK).

### 4.2. Scattering Experiments

SANS measurements were performed at D22, Institute Laue-Langevin, Grenoble, France [37]. We measured three configurations at sample-to-detector distances of 1.6, 5.6 and 17.8 m with corresponding collimations of 2.8, 5.6 and 17.8 m and a wavelength of 6 Å (Δ*λ/λ* = 10%). Data were recorded on a ^3^H multidetector of 128 linear sensitive Reuter–Stokes^®^ detector tubes, with a pixel size of 0.8 × 0.8 cm. Samples were filled in Hellma 120-QS cuvettes of 1 mm pathway and measured at 50 °C. Lipid concentrations were 5 mg/mL in 100% D_2_O, 10 mg/mL in 75% D_2_O and 15 mg/mL in 50% D_2_O. Data were reduced using GRASP (www.ill.eu/users/support-labs-infrastructure/software-scientific-tools/grasp/ accessed on 25 June 2019), performing flat field, solid angle, dead time and transmission correction, normalizing by incident flux and subtracting contributions from empty cell and solvent.

SAXS data were recorded at BM29, ESRF, Grenoble, France (Experiment MX-2282), equipped with a Pilatus3 2M detector, using a photon energy of 15 keV at a sample-to-detector distance of 2.867 m [38]. Samples were measured at a concentration of 10 mg/mL, at 50 °C and exposed 20 times for 2 s in a flow-through quartz capillary of a 1 mm light path length. Data reduction and normalization were performed by the automated ExiSAXS system; for the subtraction of the solvent and capillary contributions SAXSutilities 2 (www.saxsutilities.eu accessed on 29 October 2020) was used. Additionally, DPPC LUVs were measured using a SAXSpoint camera (Anton Paar, Graz, Austria) connected to a MetalJet X-ray generator (Excillum, Kista, Sweden) with a liquid, Ga-rich alloy, jet anode. Data were recorded using an Eiger R 1 M detector system (Dectris, Baden-Daettwil, Switzerland) and reduced via the software SAXSanalyis (Anton Paar).

### 4.3. SDP-Modeling of Lipid Bilayers

Small-angle scattering (SAS) data were analyzed in terms of a probability-density-based approach, also known as the scattering density profile (SDP) model, which is frequently used in small-angle scattering and reflectrometry, e.g., [14,39-41]. We used the same parsing scheme as Kučerka et al. [3] for saturated phosphatidylcholines, describing the volume probability distributions of individual moieties of the lipid molecules by Gaussian distributions (terminal methyls, carbonyl-glycerol backbone, phosphate group, choline-CH_3_ group) and error-functions (hydrocarbon chains without terminal methyls), see Figure 1 and Appendix A. From these functions, the neutron or X-ray scattering length density profiles can easily be calculated. The model in its current form has been applied to describe SAXS data from LUVs in the range of scattering vectors, *q* from 0.1 to 0.6 Å^−1^; lower-*q* data were excluded from the SDP analysis. This motivated us to introduce a few adjustments, permitting us to extend the *q* range by one order of magnitude.

Upon combining the SDP-model, which describes a flat bilayer, with an appropriate model to describe the overall vesicle shape—according to the separated form factor model [15], we found that the calculated intensities did not fit experimental SAXS data in the low-*q* region (Figure 1). The position of the first minimum connected to the membrane structure (see Figure 1a, *q* ~ 0.02 Å^−1^) suggests that the electron density in the head group region is higher than initially thought. One way to account for this is by introducing a layer of higher density water around the headgroup. This was inspired by previous considerations about lipid bilayers [39] as well as the established necessity to include a hydration layer in protein and nucleic acid models [26]. Hydration water was included into the model using another error-function adjacent to the ones describing the hydrocarbon chains, with the same smearing parameter *σ*_*CH*2_ and reaching up to the position of the choline-CH_3_ group in addition of *σ_Chol_*. This ensures that the hydration layer always surrounds the headgroup by roughly one water molecule. We used a width of *d_shell_* = 3.1 Å around the lipid head group and set the upper limit for the volume per molecule to the bulk water value of 30.28 Å^3^ (see Appendix B Table A4).

The second modification addresses the mismatch of the model with the depth of bilayer-related minima of the X-ray data. We were able to account for this by including a Gaussian polydispersity on the membrane thickness. It is implemented by varying only the width of the hydrocarbon chain region, while keeping all other parameters unchanged. One could attempt to extend the model to a more flexible headgroup for states of different unit cell area, however, as described in the result section. However, one would risk that area-compressed states could end up with an over-filled unit cell. Furthermore, headgroup parameters from scattering data are generally ill-defined and highly correlated; therefore we remained with a static headgroup. A possible physical explanation for this effect is the influence of peristaltic modes, which were found for this *q*-region in MD-simulations [42]. These fluctuations, however, do not exert the same amplitudes for all wavelengths. This might also explain why our implementation, despite the large improvement in fit quality, still did not perfectly match the form factor minima.

We further note that the various volume probability functions in our model do not necessarily overlap perfectly for all configurations of positions and standard deviations, potentially leading to an overfilling of the unit cell, which the model would automatically compensate for with “negative water”. To have our optimization algorithm automatically avoid these regions, we introduced a penalty on the cost function minimized in the procedure. To do this, we calculated the number of negative water molecules *n*_*H*_2_*O*_ in each iteration and modified the cost function $\chi^{2}\to \chi^{2}+n_{H_{2}O}^{2}∕\sigma_{H_{2}O}^{2}$. The strength of the penalty can be tuned by adjusting $\sigma_{H_{2}O}^{2}$.

In order to “equalize” contributions of SAXS and SANS data to fitting results, we apply a semi-empirical method to weight the cost functions ($\chi_{i}^{2}$) of all datasets, according to the examined *q*-ranges as well as the number of data points recorded, using:

(4) $$\chi^{2}=\chi_{X}^{2}+\alpha \chi_{N}^{2},$$

where $\chi_{X}^{2}$ and $\chi_{N}^{2}$ are the cost functions for X-ray and neutron scattering data, respectively. We determine the scaling coefficient *α* from the ratio of densities of points in the *q*-space:

(5) $$\alpha =\frac{n_{X}\;∕\;(q_{X}^{max}-q_{X}^{min})}{N_{N}n_{N}\;∕\;(q_{N}^{max}-q_{N}^{min})},$$

*n_X/N_* being the number of points per X-ray/neutron measurement. If there is more than one neutron contrast, we divide in addition by the number of neutron measurements *N_N_*, provided that all neutron measurements have the same density of points.

Using this approach, we examined the impact of contrast variation by changing the H_2_O/D_2_O ratio in the solvent. In the case of MSPC, SMPC and PMPC, where we measured 3 contrasts using 100%, 75% and 50% D_2_O, we found only negligible differences in the resulting parameters when fitting either all 3 or only 100% D_2_O. This is due to the dominant contrast emerging from the protiated hydrocarbon chains. Lipid headgroups are roughly contrast matched at 50% D_2_O. However, their contribution is already small at 100% D_2_O. Hence, there is only little gain in information from including the 50% and 75% D_2_O measurements. For the analysis of POPC, SOPC and MSM, we therefore only used one SANS-contrast.

Parameter optimization was performed using the Trust Region Reflective algorithm from the SciPy 1.6.2 package [43]. To analyze parameter correlations within the model, we used the No-U-Turn Sampler within the PyMC3 package [44,45].

### 4.4. Molecular Dynamics Simulations

At the time of bilayer construction, the three lipids, MSPC, PMPC and SMPC, were not available in the CHARMM-GUI web server [46-50]. We therefore first used CHARMM-GUI to construct the bilayers of pure distearoyl PC (DSPC) or pure DPPC lipids. Each bilayer had 100 lipids per leaflet (200 lipids total) and was hydrated with 45 water molecules per lipid (without any salt ions). PMPC was then built from the DPPC bilayer by removing the last carbon on the *sn1* chain (C216 in CHARMM36 notation) together with its 3 hydrogens (H16R, H16S, H16T) and the 2 hydrogens bonded to the last-but-one carbon on that same chain (H15R and H15S). Carbon C215 was then changed to hydrogen (H14T) by modifying its atom name, type and charge accordingly to complete the terminal methyl group of the myristoyl chain of the newly created PMPC lipid.

The MSPC and SMPC bilayers were similarly generated from the DSPC bilayer by removing the last 3 carbons and their hydrogens on the *sn1* or *sn2* chains, respectively, then modifying the 15th carbon by removing its hydrogens and changing its name, type and charge to complete the terminal methyl group of the myristoyl chain of the newly created lipids. Additionally, a pure DMPC bilayer was constructed with CHARMM-GUI. The bilayer had 100 lipids per leaflet and was hydrated with 45 water molecules per lipid.

All simulations were run with the NAMD software [51] and the CHARMM36 force field for lipids [52,53]. Each of the bilayer systems, excluding DMPC, was energy minimized for 1200 steps, then simulated for a total of 1 ns with an integration time-step of 1 fs before the production run which employed a time-step of 2 fs. DMPC was equilibrated following CHARMM-GUI’s 6-step equilibration protocol. All simulations were run at a constant temperature of 50 °C (323K) and a pressure of 1 atm maintained by NAMD’s Langevin thermostat and Nose–Hoover Langevin piston, respectively. Long-range interactions were modeled with a 10–12 Å Lennard-Jones potential using NAMD’s vdwForceSwitching option. All hydrogen bonds were constrained with the rigidbonds parameter set to all and electrostatic interactions were modeled using the particle mesh Ewald (PME) method with a grid spacing of 1 Å. The four simulations were run for a total of 1 μs (MSPC), 0.969 μs (PMPC), 1.03 μs (SMPC) and 0.8 μs (DMPC). The first 50 ns were discarded and the rest were used to calculate the number density profile of each system with the density plugin in VMD [54]. The calculation was done at a resolution (slab thickness) of 0.2 Å on trajectory frames spaced 100 ps apart. For comparison, a system of a DPPC bilayer simulated under the same conditions was taken from [55] and its number density profile was calculated following the same procedure.

The volumes of water molecules from the simulations were calculated with the Voro++ software library (http://math.lbl.gov/voro++/ accessed on 15 July 2021). Briefly, the indices and coordinates of all atoms in a trajectory frame were used as input to Voro++ which partitioned the space into a discrete number of 3-dimensional Voronoi cells by taking into account the periodic images of the simulation box. The resulting volumes of the water atoms were then extracted, properly grouped to obtain the volumes of the individual water molecules, and binned according to their z positions in MATLAB. The results from all frames were averaged to produce the final plot of water volume as a function of z.

## Data Availability Statement:

SANS data is available from ref. [37]. All other data can be obtained from the authors upon reasonable request.

## Supplementary Material

1

## Figures and Tables

**Table 1.** Results from joint SAXS/SANS data analysis/from MD simulations: Area per lipid *A*, Luzzati thickness *D_B_*, hydrophobic thickness 2*D_C_*, standard deviation of the terminal methyl Gaussian *σ*_*CH*3_, relative methyl overlap *ϒ* (dimensionless). The column *e* gives an error estimate relative to the values in the table.

	*ϵ* (%)	DPPC	MSPC	SMPC	PMPC	POPC	SOPC	MSM
*A* [Å^2^]	2	63.1	62.2	62.0	62.9	67.5	68.8	64.8
*D_B_* [Å]	5	40.3	40.3	40.3	38.4	38.4	39.4	42.1
2*D_C_* [Å]	3	28.6	29.1	29.2	27.0	28.4	29.2	32.8
*Å*_*CH*3_ [Å]	5	2.91	3.34	3.67	3.12	3.41	3.31	4.29
*n_W_*	10	9.7	11.3	12.8	12.1	16.6	15.1	9.6
*ϒ*	7	0.43	0.55	0.65	0.54	0.60	0.55	0.71

## Appendix

### Appendix A. Full SAS-Model

The signal in small-angle scattering is described by the absolute square of the form factor, meaning the Fourier-transform of the scattering length density profile (SDP). As the overall vesicle shape and the trans-bilayer structure contribute on different length scales, we can describe them separately and approximate the bilayer as an infinite flat sheet [15]. As we are using error-functions and Gaussians to describe the SDP, the required Fourier transforms are given in the following. Note that the formulas omit the imaginary part of the form factor, which is antisymmetric around the origin and therefore vanishes for a symmetric trans-bilayer profile.

**Algorithm A1:** Iterative fitting of the chain length mismatch correction to a linear function Parameters behind; in function definitions designate fixed inputs in the optimizations Data inputs are Δ*l_C_*, *σ*_*CH*3_

$$\begin{array}{cc} \; & \;\mathrm{function}\;Flin(k,\sigma_{CH3}^{sym};\;|\Delta l_{C,corr}|)\\ \; & \;\\ \; & \;\;\;\mathrm{return}\;k|\Delta l_{C,corr}|+\sigma_{CH3}^{sym}\\ \; & \;\\ \; & \;\mathrm{function}\;Fcorr(d_{tilt};\;\Delta l_{C},k,\sigma_{CH3}^{sym})\\ \; & \;\\ \; & \;\;\;|\Delta l_{C,corr}|\leftarrow |\Delta l_{C}+d_{tilt}|\\ \; & \;\\ \; & \;\;\;\mathrm{retun}\;k|\Delta l_{C,corr}|+\sigma_{CH3}^{sym}\\ \; & \;\\ \; & \;\mathrm{initialize}:k,\sigma_{CH3}^{sym},d_{tilt},\;|\Delta l_{C,corr}|\\ \; & \;\\ \; & \;\mathrm{while}\;|Flin-Fcorr|>\varepsilon \;\mathrm{do}\\ \; & \;\\ \; & \;\;\;k,\sigma_{CH3}^{sym}\leftarrow \text{optimize}\;Flin-\sigma_{CH3}=0\\ \; & \;\\ \; & \;\;\;d_{tilt}\leftarrow \text{optimize}\;Fcorr-\sigma_{CH3}=0\\ \; & \;\\ \; & \;\mathrm{end}\;\mathrm{while} \end{array}$$

The real part of the Fourier-transform for a slab, described by two mirrored error-functions centered around *μ*, with a width of *d*, a smearing parameter of *σ* and its area normalized to 1, is given by

(A1)
$$ℜ\left\{\frac{1}{2d}\int_{-\infty }^{\infty }\left[\text{erf}\left(\frac{x-\mu +d∕2}{\sqrt{2}\sigma }\right)-\text{erf}\left(\frac{x-\mu -d∕2}{\sqrt{2}\sigma }\right)\right]e^{iqx}dx\right\}=\frac{\sin(qd∕2)}{qd∕2}e^{-\frac{\sigma^{2}q^{2}}{2}}\;\cos(\mu q)$$

For the Gaussian distribution centered at *μ* and standard deviation *σ* we use the following:

(A2)
$$ℜ\left\{\int_{-\infty }^{\infty }\frac{1}{\sqrt{2\pi }\sigma }e^{-\frac{(x-\mu {)}^{2}}{2\sigma^{2}}}e^{iqx}dx\right\}=e^{-\frac{q^{2}\sigma^{2}}{2}}\;\cos(q\mu )$$

**Table A1.** Molecular groups described by individual functions * Sphingosine backbone in the case of MSM.

Abbr.	Content	Function
CH3	Terminal methyl group	Gaussian
CH2	Methylene chains	Error function
CG	Carbonyl–glycerol backbone *	Gaussian
PCN	Phosphate + CN	Gaussian
Chol	Choline-CH3 group	Gaussian
BW	Hydration layer	Error function

We added up scattering contributions of the parts in Table A1 by using the normalized functions (A1) and (A2), weighted by the factors $\frac{V_{k}}{A}$, *A* denoting the area per lipid and *V_k_* the volume of the respective moiety. The functions for CH2 and BW are treated differently: they are normalized to fill the whole unit cell area, followed by the subtraction of the groups they contain. We applied polydispersity on the chain-width *D*_*C*_ by summing over a series of form factors with different *D*_*C,i*_, weighted by a Gaussian distribution $N(x|{\bar{D}}_{C},\sigma_{poly})$ with a mean ${\bar{D}}_{C}$ and standard deviation *σ_poly_*. The average chain-width is calculated by ${\bar{D}}_{C}=\frac{n_{CH2}V_{CH2}+2V_{CH3}}{A}$. Contrasts of the individual moieties *k* are defined by $\Delta \rho_{k}=\frac{b_{k}}{V_{k}}-\rho_{solvent}$, *b* and *ρ* denoting scattering length and scattering length density for either radiation (X-rays or neutrons). A graphical representation of all distances between moieties and thicknesses is given in Figure A1:

I(q)∝Fsphere(rmean,σR)∑iN(DC,i∣D¯C,σPoly)[]2(ΔρT−ΔρCH2)VCH3DC,iVCH2+VCH3e−q2σCH322+2ΔρCH21qe−q2σCH222sin(qDC,i)+2(ΔρCG−ΔρBW)VCGAe−q2σCG22cos(q(DC,i+dCG∕2))+2(ΔρPCN−ΔρBW)VPCNAe−q2σPCN22cos(q(DC,i+dCG+dPCN∕2))+2(ΔρChol−ΔρBW)VCholAe−q2σChol22cos(q(DC,i+dCG+dPCN+dChol∕2))+4ΔρBW1qe−q2σCH222sin(qdCG+dPCN+dChol+dshell2)cos(q(DC,i+dCG+dPCN+dChol+dshell2))[]2+Iinc

To describe the contribution from the overall vesicle shape, we use the Schultz-distributed form factor of a sphere, as described in Kucerka et al. 2007 [56]:

$$F_{sphere}=\frac{8\pi^{2}(z+1)(z+2)}{s^{2}q^{2}}\left\{1-{\left(1+\frac{4q^{2}}{s^{2}}\right)}^{-(z+3)∕2}\cos\left[(z+3)\;\text{arctan}\left(\frac{2q}{s}\right)\right]\right\}$$

Mean vesicle radius *R*_*m*_ and polydispersity *σ*_*R*_ come in via the auxiliary quantities *s* and *z*:

$$s=\frac{R_{m}}{\sigma_{R}^{2}},\;\;z=\frac{R_{m}^{2}}{\sigma_{R}^{2}}-1$$

### Appendix B. SDP-Model Parameters

Tables A2 and A3 contain all information about the SDP-profiles for all studied lipids and references. Parameter notation was chosen to conform to former publications such as [3].

**Table A2.** Results from joint SAXS-SANS analysis of LUVs containing saturated lipids, in comparison to literature values and simulations. *ϵ* in the second column denotes relative error-estimates from our SAS-experiments. Quantities not marked with any symbol (*, ^†^, ^‡^) were adjustable during the analysis.

	*ϵ* (%)	DPPC ^a^	DPPC ^b^	DPPC ^c^	MSPC ^a^	MSPC ^c^	SMPC ^a^	SMPC ^c^	PMPC ^a^	PMPC ^c^
$V_{L}^{*}({Å}^{3})$		1232	1228.5	1209.2	1232	1210	1232	1211.1	1175.8	1155.7
$V_{H}^{*}({Å}^{3})$		328	331	314.4	328	314.6	328	315.4	328	314.7
$r_{CG}^{*}$		0.44	0.40	0.48	0.44	0.48	0.44	0.49	0.44	0.48
$r_{PCN}^{*}$		0.3	0.29	0.21	0.3	0.22	0.3	0.22	0.3	0.22
*r* *		2.09	1.95	2.06	2.09	2.06	2.09	2.06	2.09	2.05
$D_{B}^{‡}(Å)$	5	40.3	38.9	39.3	40.3	39.1	40.3	38.2	38.4	36.6
$D_{HH}^{‡}(Å)$	3	37.5	38.4	38.4	35.7	38.4	34.8	37.6	33.9	36
$2D_{C}^{‡}(Å)$	3	28.6	28.4	29.1	29.1	28.9	29.2	28.3	27.0	26.6
$D_{H1}^{‡}(Å)$	20	4.5	4.97	4.7	3.3	4.8	2.8	4.7	3.5	4.7
*A* (Å^2^)	2	63.1	63.1	61.6	62.2	62	62.0	63.4	62.9	63.2
*z_CG_* (Å)	8	15.2	14.7	16.4	15.6	16.2	15.7	15.9	14.5	15
*σ_CG_* (Å)	20	2.5	2.19	2.93	2.5	2.97	2.5	2.99	2.5	2.85
*z*_*PCN*_ (Å)	8	19.2	19.6	20.1	18.7	19.9	18.4	19.6	17.8	18.7
*σ_PCN_* (Å)	20	2.3	2.35	2.99	3.1	3.04	3.1	3.06	3.0	2.92
*z*_*Chol*_ (Å)	3	21.1	20.2	21.39	22.3	21.2	23.1	20.89	21.5	20.1
$\sigma_{Chol}^{†}(Å)$		3	2.98	3.6	3	3.63	3	3.63	3	3.51
$\sigma_{CH2}^{†}(Å)$		2.5	2.47	2.83	2.5	2.88	2.5	2.88	2.5	2.73
σ_*CH*3_ (Å)	5	2.9	2.94	3.23	3.3	3.59	3.7	4.32	3.1	3.58
*σ_poly_* (%)	6	3.6	0		2.9		5.3		3.5	
$V_{W,bound}^{‡}$	6	29.3			29.3		29.3		29.3	
(Å^3^)										
$n_{W}^{‡}$	10	9.7			11.3		12.8		12.1	
*ϒ* ^‡^	7	0.43	0.46	0.52	0.55	0.63	0.65	0.88	0.54	0.71

a SAS—analyis, this work

b Kučerka et al. [3]

c MD—simulations, this work

* fixed according to Nagle et al. [30]

† fixed

‡ calculated quantity.

**Table A3.** Results from joint SAXS-SANS analyis of LUVs containing unsaturated lipids and comparison to literature values. *ϵ* in the second column denotes relative error-estimates from our SAS experiments. Quantities not marked with any symbol (*, ^†^, ^‡^) were adjustable during the analysis.

		*ϵ* (%)	POPC ^a^	POPC ^b^	SOPC ^a^	SOPC ^b^	MSM ^a^
$V_{L}^{*}({Å}^{3})$	Lipid volume		1276.9	1275.5	1333.1	1327.5	1336.3
$V_{H}^{*}({Å}^{3})$	Headgroup volume		320	331	328	331	274
$r_{CG}^{*}$	*V_CG_/V_H_*		0.45	0.41	0.44	0.43	0.32
$r_{PCN}^{*}$	*V_PCN_/V_H_*		0.29	0.3	0.3	0.3	0.32
*r* *	*V*_*CH*3_ */V*_*CH*2_		2.09	1.93	2.09	1.94	2.09
$r_{12}^{*}$	*V_CH_/V*_*CH*2_		0.8	0.8	0.8	0.8	0.8
$D_{B}^{‡}(Å)$	Luzzati bilayer thickness	5	38.4	37.9	39.4	39.0	42.1
$D_{HH}^{‡}(Å)$	Head–head distance	3	37.5	35.9	35.7	37.0	43.0
$2D_{C}^{‡}(Å)$	Hydrophobic thickness	3	28.4	28.1	29.2	29.3	32.8
$D_{H1}^{‡}(Å)$	(*D_HH_* – 2*D_C_*)/2	20	4.6	3.91	3.3	3.9	5.1
*A* (Å^2^)	Area per lipid	2	67.5	67.3	68.8	68.1	64.8
*z*_*CG*_ (Å)	*D_C_* + *d_CG_*/2	8	15.0	14.8	15.9	15.5	18.4
*σ_CG_* (Å)		20	2.5	2.48	2.5	2.5	2.5
*z_PCN_* (Å)	*D*_*C,i*_ + *d_CG_* + *d_PCN_*/2	8	19.1	19.3	19.0	19.5	22.1
*σPCN* (Å)		20	2.5	2.81	3.0	2.7	2.4
*z*_*Chol*_ (Å)	*D*_*C*_ + *d*_*CG*_ + *d*_*PCN*_ + *d*_*Chol*_/2	3	23.4	20.3	23.0	20.5	22.1
$\sigma_{Chol}^{†}(Å)$			3	2.98	3	2.98	3
$\sigma_{CH2}^{†}(Å)$			2.5	2.50	2.5	2.5	2.5
*σ*_*CH*3_ (Å)		5	3.4	2.69	3.3	3.1	4.3
*σ_poly_* (%)	Thickness polydispersity	6	7.9	0	3.6	0	3.5
$V_{W,bound}^{‡}({Å}^{3})$	Volume per bound water molecule	6	29.9		29.7		29.8
$n_{W}^{‡}$	Number of bound waters	10	16.6		15.1		9.6
*ϒ* ^‡^	Relative methyl overlap	7	0.60	0.41	0.55	0.50	0.71

a SAS—analyis, this work

b Kučerka et al. [3];

c MD—simulations, this work,

* fixed according to Nagle et al. [30],

† fixed,

‡ calculated quantity.

The volume of MSM was measured via the vibrating tube principle [57] using a DMA 4500 M density meter (Anton Paar). We measured the density *ρ_s_* of 3 concentrations of MSM at 50 °C, prepared as described in Section 4.1 in H_2_O without extruding (Table A4). The volume per MSM molecule was calculated by the following Equation [58], using the lipid molecular weight *M_L_* = 785.034 g/mol, masses of water *m_w_* and lipid *m_L_* according to the concentrations given in Table A4, and a water density *ρ*_*w*_ of 0.98806 g/mL. The density measurements were performed with a nominal accuracy of 0.00005 g/mL:

(A3)
$$V_{L}=\frac{M_{L}}{0.6022\rho_{s}}\left[1+\frac{m_{w}}{m_{L}}\left(1-\frac{\rho_{s}}{\rho_{w}}\right)\right],$$

**Table A4.** Volumetric measurements of MSM vesicles in H_2_O. *c*… concentration of lipid. *ρ*_*s*_… measured density. *V_L_*… volume per MSM molecule according to Equation (A3).

*c* (g/L)	*ρ*_*s*_ (g/mL)	*V_L_* (Å^3^)
10	0.98798	1330
5	0.98803	1327
2.5	0.98800	1351
0	0.98806	(pure H_2_O)
	average	1336 ± 15

### Appendix C. Evaluation of *σ*_*CH*3_-Data

**Table A5.** Chain lengths *D_C_* at 50 °C in chain-symmetric phosphatidylcholines from previous scattering studies.

Chain	*D_C_*	Reference
14:0	12.4	[3]
16:0	14.3	[3]
18:0	16.2	[3], extrapolated
22:0	20.1	[3], extrapolated
18:1	13.0	[59], extrapolated
PSM	13.3	[34], 45 °C

## Abbreviations

The following abbreviations are used in this manuscript:

SAXS: Small-angle X-ray scattering
SANS: Small-angle neutron scattering
SLD: Scattering length density
SDP: Scattering density profile
MD: Molecular dynamics
MCMC: Markov chain Monte Carlo
LUV: Large unilamellar vesicle
DPPC: 1,2-dipalmitoyl-sn-glycero-3-phosphocholine
MSPC: 1-myristoyl-2-stearoyl-sn-glycero-3-phosphocholine
SMPC: 1-stearoyl-2-myristoyl-sn-glycero-3-phosphocholine
PMPC: 1-palmitoyl-2-myristoyl-sn-glycero-3-phosphocholine
MSM: Milk sphingomyelin
