# Supplementary material for: Structure and Interdigitation of Chain-Asymmetric Phosphatidylcholines and Milk Sphingomyelin in the Fluid Phase
Source: Symmetry (Basel). Author manuscript; Available in PMC 2022 May 6. (PMC9075682; doi:10.3390/sym13081441)
Supplement: 1 [file NIHMS1749599-supplement-1.pdf]

Appendix D. Supplementary Figures

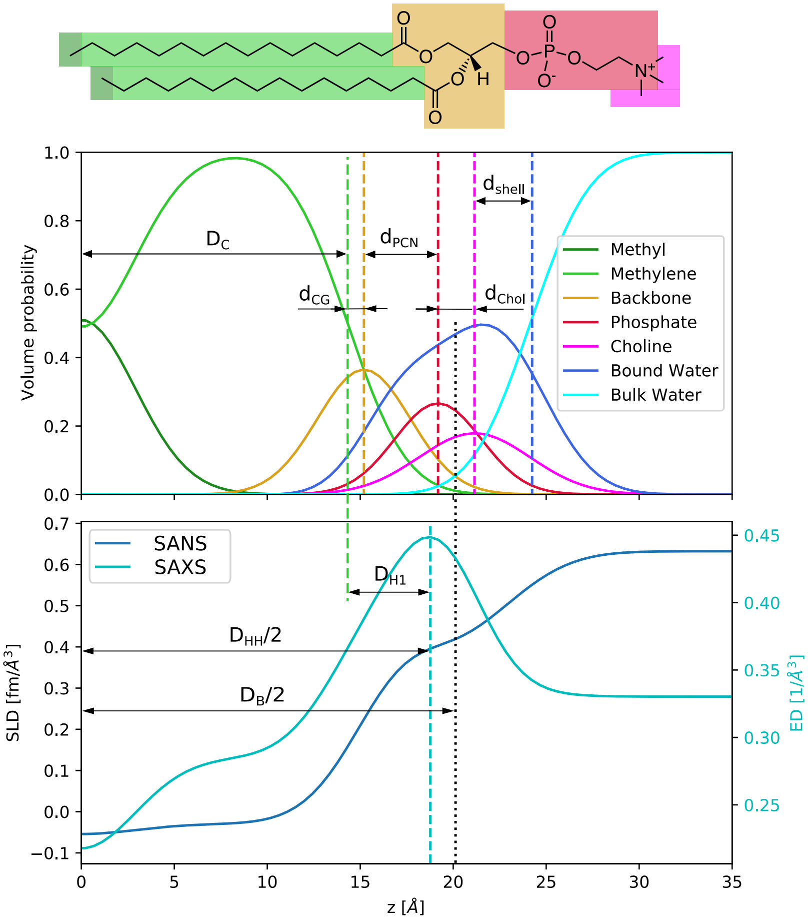

Figure A1. Examplary profile of probability distribution functions (**top**) and scattering length density (SLD)/electron density (ED) profiles (**bottom**) with definitions of distances used in the SDP-model.

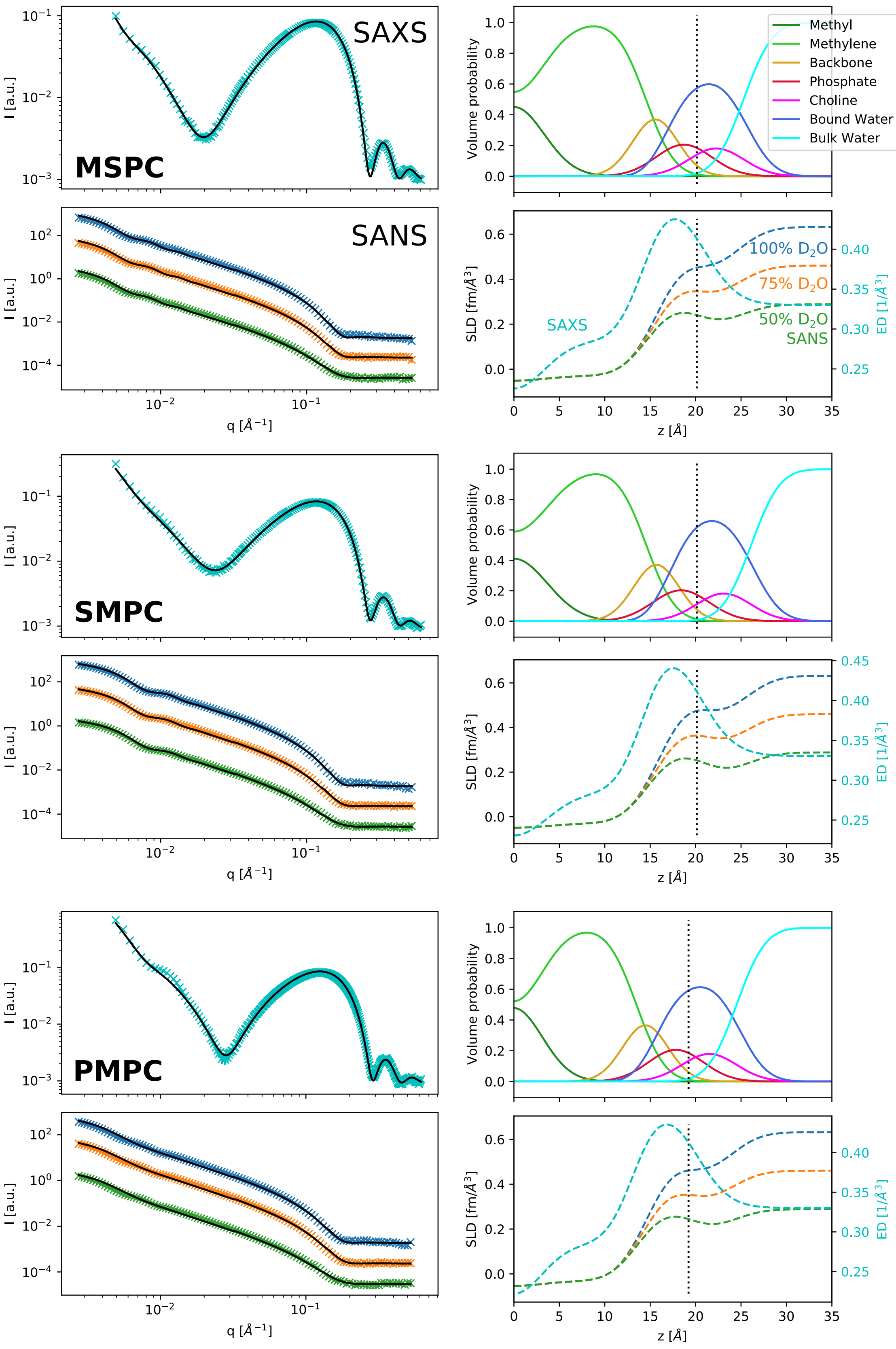

Figure A2. SAXS and SANS data with fits (black lines); SDP-volume probability, electron density and neutron scattering length density profiles for MSPC, SMPC and PMPC. Neutron intensities of different contrasts have been shifted for better visibility.

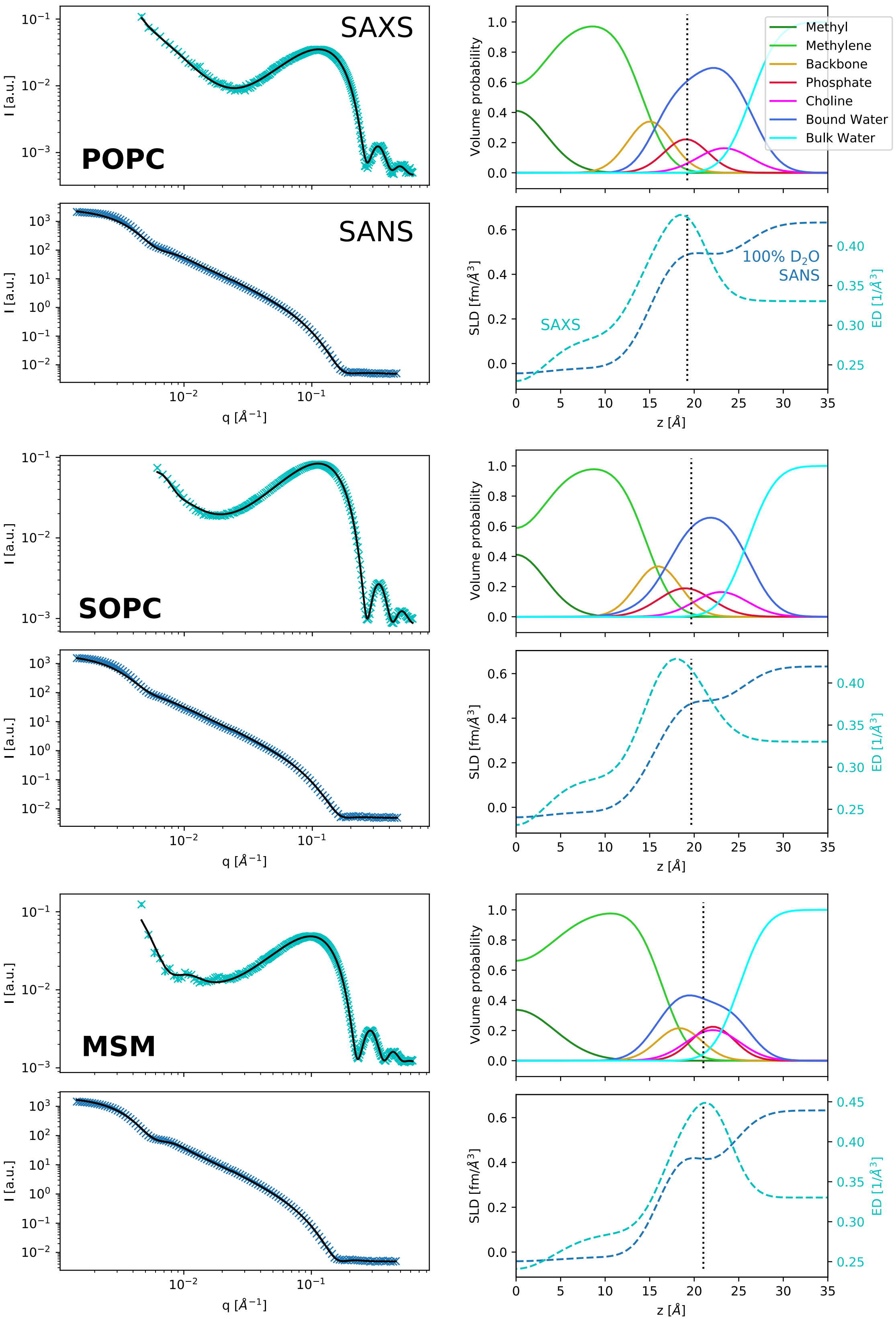

Figure A3. SAXS and SANS data with fits (black lines); SDP volume probability, electron density and neutron scattering length density profiles for POPC, SOPC and MSM.

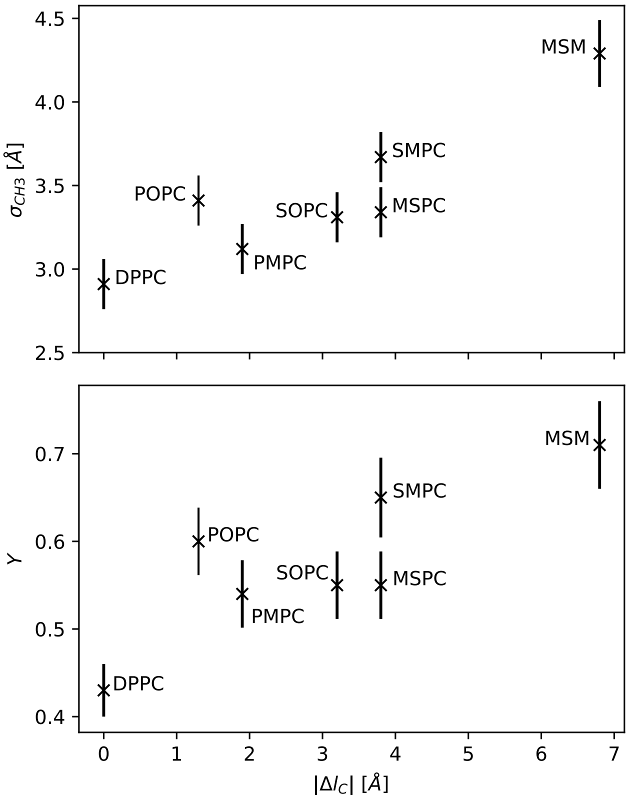

Figure A4. Standard deviations σ_{CH3} of the Gaussian volume distributions of the terminal methyl groups (upper plot) and relative interdigitation parameters (lower plot), plotted over the chain length mismatch Δl_C of the respective lipids.

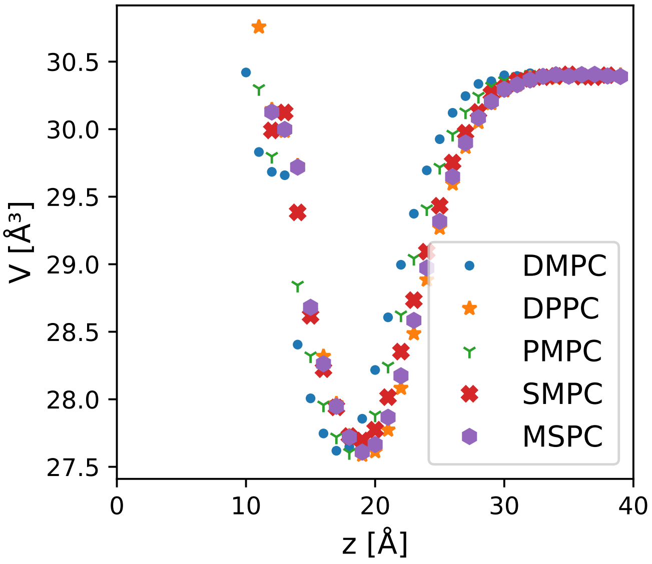

Figure A5. Volume per water molecule across the bilayer calculated from MD simulations. Regions without data points (notably for $z < 10$ Å) do not contain water molecules.

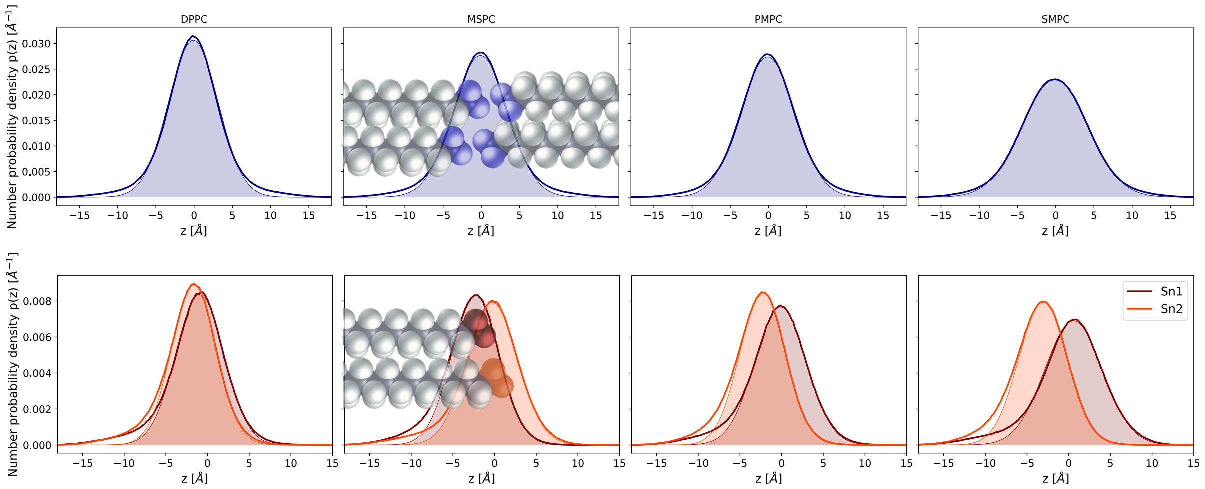

Figure A6. The upper panels show the total number probability density distributions of all CH_3 groups in the bilayer for DPPC and the chain-asymmetric saturated lipids MSPC, PMPC and SMPC. Shaded areas and lightly drawn lines correspond to Gaussian functions fitted to the distribution. In the lower panels, the distributions are divided into the CH_3 groups of *sn1* and *sn2* chains, showing just the lipids from the left side of the bilayer. Again, Gaussians are inserted in the form of shaded areas. They fit almost perfectly in all cases on the right side of the distributions, however, to the left, there is some mismatch, which also causes the mismatch of the overall distribution. This might be caused by chains bending back towards their headgroups.

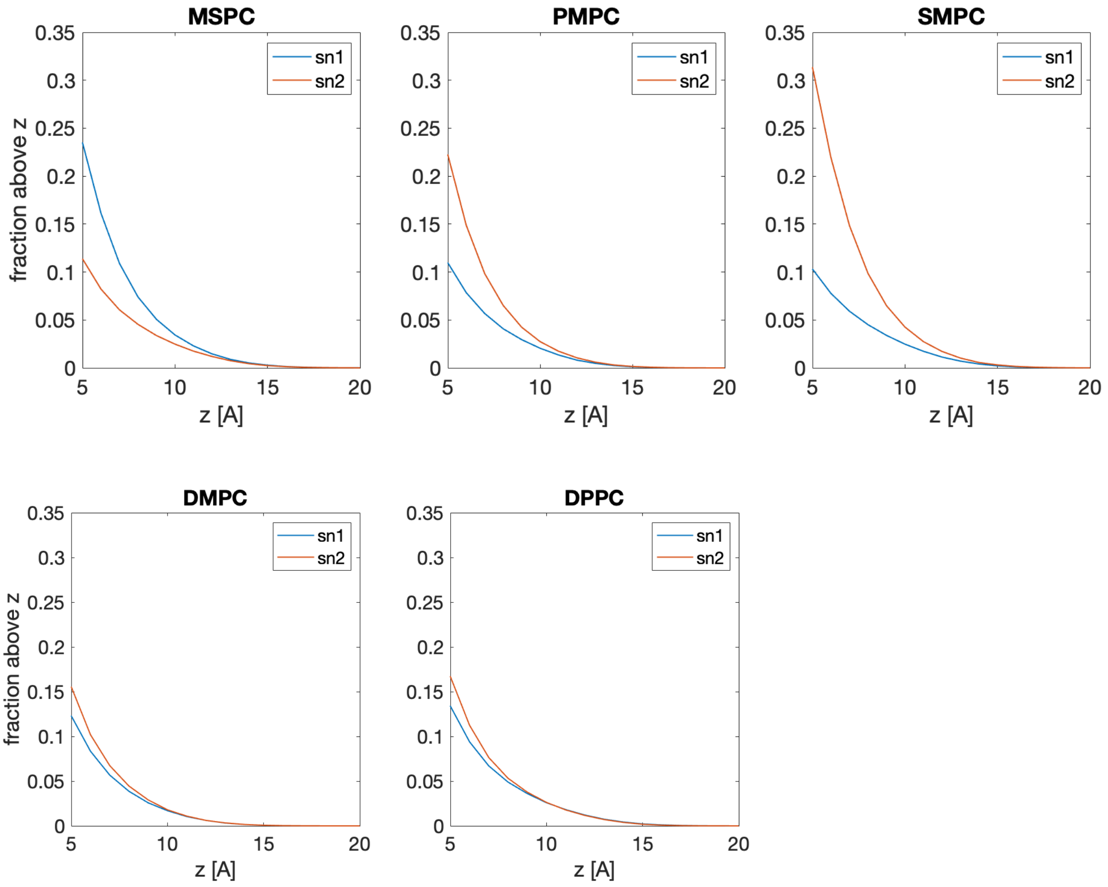

Figure A7. Fraction of lipids whose terminal methyl group is above a certain z position. The fraction was calculated at every point in time (i.e., in each frame of the simulation trajectory) and is averaged over all frames. The bilayer midplane in all bilayers is at z = 0. The data show that more lipids bend their 14:0 chains in the chain-asymmetric bilayers while both chains bend to a similar extent in the chain-symmetric bilayers.
